# Electronic and Vibrational Properties of Allene Carotenoids

**DOI:** 10.1021/acs.jpca.1c09393

**Published:** 2022-02-03

**Authors:** Mindaugas Macernis, Simona Streckaite, Radek Litvin, Andrew A. Pascal, Manuel J. Llansola-Portoles, Bruno Robert, Leonas Valkunas

**Affiliations:** † Institute of Chemical Physics, Faculty of Physics, Vilnius University, Saulėtekio Avenue 3, LT-10222 Vilnius, Lithuania; ‡ Institute for Integrative Biology of the Cell, CEA, CNRS, Université Paris-Saclay, 91198 Gif-sur-Yvette, France; § Biology Centre, Czech Academy of Sciences, Branisovska 31, 370 05 Ceske Budejovice, Czech Republic; ∥ Faculty of Science, University of South Bohemia, Branisovska 1760, 370 05 Ceske Budejovice, Czech Republic; ⊥ Molecular Compounds Physics Department, Center for Physical Sciences and Technology, Sauletekio Avenue 3, LT-10257 Vilnius, Lithuania

## Abstract

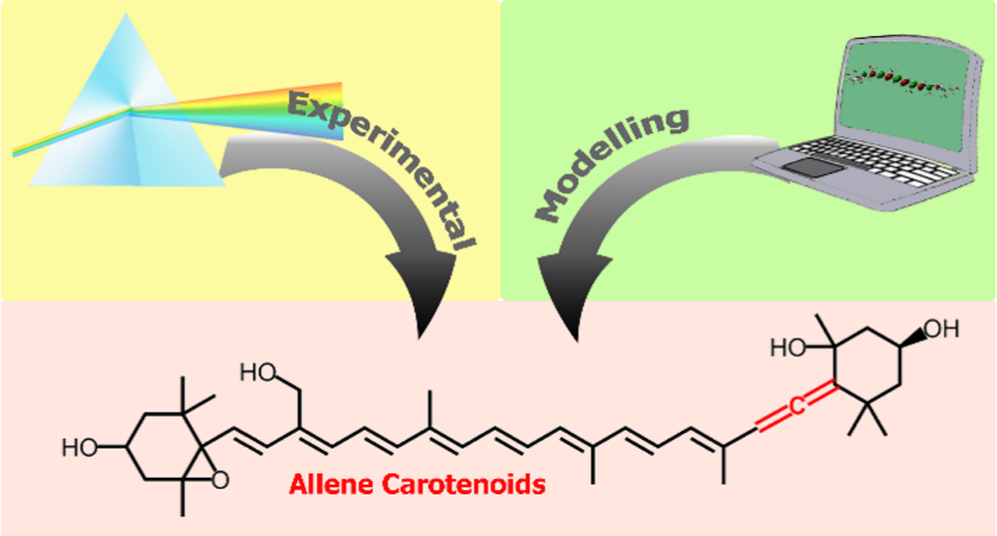

Carotenoids are conjugated linear molecules built from the repetition of terpene units, which display a large structural diversity in nature. They may, in particular, contain several types of side or end groups, which tune their functional properties, such as absorption position and photochemistry. We report here a detailed experimental study of the absorption and vibrational properties of allene-containing carotenoids, together with an extensive modeling of these experimental data. Our calculations can satisfactorily explain the electronic properties of vaucheriaxanthin, where the allene group introduces the equivalent of one C=C double bond into the conjugated C=C chain. The position of the electronic absorption of fucoxanthin and butanoyloxyfucoxanthin requires long-range corrections to be found correctly on the red side of that of vaucheriaxanthin; however, these corrections tend to overestimate the effect of the conjugated and nonconjugated C=O groups in these molecules. We show that the resonance Raman spectra of these carotenoids are largely perturbed by the presence of the allene group, with the two major Raman contributions split into two components. These perturbations are satisfactorily explained by modeling, through a gain in the Raman intensity of the C=C antisymmetric stretching mode, induced by the presence of the allene group in the carotenoid C=C chain.

## Introduction

Carotenoids are conjugated linear molecules built from the repetition of terpene units, which perform a wide variety of functions in biology. Their linear conjugated isoprenoid chain affords them an intense absorption in the blue–green range, and the colors they confer on fruits, flowers, and animals are at the basis of complex signaling processes. In photosynthetic organisms, they are implicated in the harvesting of solar photons in a spectral region, where chlorophyll possesses only minimal absorption, while also playing major roles in photoprotection. Natural carotenoids display a large structural diversity, and more than 1100 molecular species have now been identified.^1^ Their carbon skeletons may include functional groups, which may or may not be conjugated with the isoprenoid chain. Taking into account the molecular C_2h_ symmetry of the simplest carotenoids, a model involving three low-energy excited states is able to account for their electronic properties.^2^ The strong carotenoid electronic absorption corresponds to a transition from the ground state to the second excited state (S_0_ → S_2_). This transition generally displays three strong vibronic components, and, for simplicity, we will refer to the energy of this transition by that of its lowest vibronic sublevel (0–0). According to polyene symmetry models, the first excited state exhibits the same symmetry as the ground state, and the optical transition between these states is thus one-photon forbidden.^3^

Simple linear carotenoids, which contain only tetraterpene units, display an elegant relationship between five structural and photophysical parameters, namely, the number of conjugated double bonds in the carotenoid chain (N), the energy of the 0–0 sublevel of their S_0_/S_2_ electronic transition, the vibrational frequency of their C=C symmetric stretching mode (ν_1_), and the energy and decay rate of their first excited state (S_1_).^2,4^ Any random pair of these five parameters (*N*, ν_1_, S_0_/S_2_ energy, S_0_/S_1_ energy, and S_1_ decay rate) allows the precise calculation of the values of the other three, as can be predicted directly from the simple C_2h_-derived model. However, there are increasing indications that some entire classes of carotenoids exhibit structures, which do not fully comply with the C_2h_ archetype, and revised models are needed to describe them. Carotenoids possessing conjugated cycles generally display properties corresponding to a shorter number of conjugated carbons that they nominally possess. We showed that these cycles are generally in a partial out-of-plane configuration, and consequently, they contribute only partially to the conjugation length of the molecule. A new parameter was defined, the effective conjugation length (*N*_eff_), to account for the partial conjugation of the end rings. For lutein and β-carotene, each conjugated cycle accounts for the equivalent of 0.3 C=C.^5^ The introduction of *N*_eff_ in the description of these carotenoids enabled an understanding of the absorption properties of lutein and β-carotene in a number of photosynthetic complexes.^6−10^ More recently, we also demonstrated that carotenoids containing a conjugated alkyne group adopt a distribution of configurations, one of which drags the conjugated cycle connected by the alkyne out of the C=C plane.^11^ These out-of-plane distortions, observed in two families of carotenoid molecules, influence the electronic properties of these molecules. It is worth noting that these nonplanar configurations may also play a role in tuning the energy required to bind these carotenoids to their protein-binding sites. They may be responsible, at least in part, for the high selectivity of these binding sites for a specific carotenoid species.

**Figure 1:**
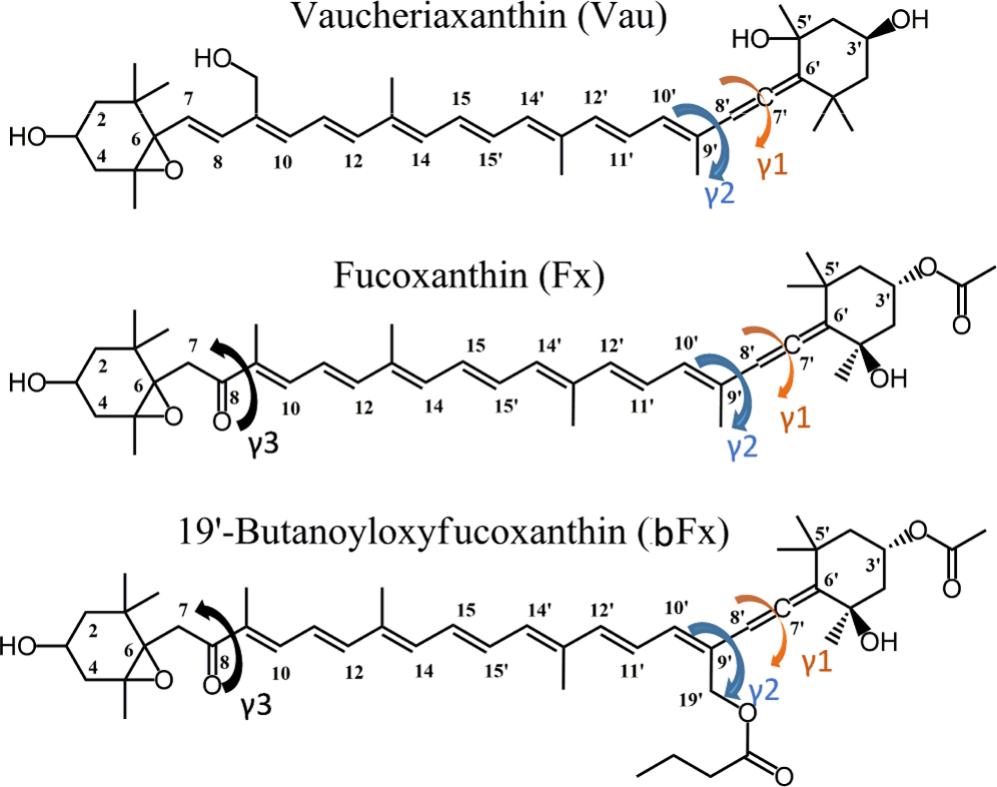
Molecular structures of the carotenoids studied in this paper.

Providing an even more complex view of carotenoid properties, the group of Polívka recently showed that nonconjugated chemical groups may play a role in influencing the photophysics of these molecules, in particular, by tuning the properties of their excited states.^12^ This was observed for the carotenoid fucoxanthin (Fx), the structure of which includes both an allene group at position C8′ and a conjugated keto group at C8 (Figure 1). The electronic properties of Fx can be tuned by its environment to a greater extent than usually observed for carotenoids, its absorption being shifted to wavelengths as high as 550 nm in photosynthetic light-harvesting proteins.^13^ This red absorption shift was proposed to arise from an intramolecular charge transfer (ICT) character of the second excited state, generated by the electron-rich keto group.^13−17^ Polívka’s group showed that this ICT can be deactivated by the presence of a nonconjugated, oxygen-rich acyloxy group at the opposite end of the conjugated chain to the keto carbonyl^12^ (see the bFx structure in Figure 1), while when positioned on the same end as the keto, this group rather enhances the ICT state.^18^ The significant electron-withdrawing character of the acyloxy group, although nonconjugated, is thus able to counteract the effect of the conjugated keto group.

In this paper, we aimed to further study the potential role of partially- or nonconjugated groups on the carotenoid structure and photophysical properties, using a combination of Raman and absorption spectroscopy and density functional theory (DFT) modeling, including Car–Parrinello molecular dynamics (CPMD) simulations. Modeling large conjugated molecules with precision generally represents a complex task and requires extensive use of DFT approaches, particularly when looking for secondary effects on the molecular electronic properties. Here, we consider three carotenoid molecules containing an allene group, whose structure is shown in Figure 1— vaucheriaxanthin (Vau), fucoxanthin (Fx), and butanoyloxyfucoxanthin (bFx). We also studied another derivative from fucoxanthin (hexanoyloxyfucoxanthin) but the results obtained were the same as for bFx. These data will thus not be reported here. These molecules were chosen because of their importance in biology. Both Fx and Vau are carotenoids synthesized by different classes of algae. They play a major role in the early steps of the photosynthetic process in these algal classes and therefore make a significant contribution to primary production on our planet. Vau possesses an allene group in position C8′, and this allene group represents the only difference in the conjugated chain relative to that of violaxanthin (Vio). In Fx and bFx, an additional C=O is present in position C8, while bFx also possesses an acyloxy group at position C19′.

## Materials and Methods

### Pigment Purification

Vau was purified from cells of marine alga *Nannochloropsis oceanica* (strain CCALA 978).^43^ Fx was purified from cells of the marine diatom *Phaeodactylum tricornutum* (strain SAG 1090-1a).^11^ bFx was purified from cells of marine alga *Aureococcus anophagefferens* (strain CCMP 1984).^44^ Sample preparation was performed in the dark, on ice. Algae cells were separated from the culture medium by centrifugation (7000*g*, 5 min), and the pigments were extracted in three solubilization steps—in methanol twice and finally acetone. In each step, the pellet was suspended in the solvent and sonicated to induce pigment release. The extracts were then pooled and dried under vacuum before dissolving in methanol prior to purification. The pigments were purified using an HPLC system, consisting of a Delta 600 pump controller, a manual injection system, and a PDA 2996 detector (Waters). The pigments were separated on a reverse-phase Zorbax SB-C18 column (4.6 × 150 mm^2^, 5 μm, silica-based, non-endcapped; Agilent), using a linear elution gradient at 1 mL·min^–1^ flow rate. A ternary solvent system was used as follows: 0–4 min linear gradient from 100% solvent-A to 100% solvent-B, 4–18 min linear gradient from 100% B to 20% B/80% C (solvent-A—80:20 methanol/0.5 M ammonium acetate (aq., pH 7.2 v/v); solvent-B—90:10 acetonitrile/water; solvent-C—100% ethyl acetate).^45^ The pigments were identified based on their absorption spectra and retention times. The peaks of interest were collected, dried out in the dark under vacuum, and stored at −80 °C. The purity of the final pigment preparation was verified by HPLC using the same protocol.^45^

### UV–Vis Absorption

Absorption spectra were measured using a Varian Cary E5 Double-beam scanning spectrophotometer (Agilent) with a 1.0 cm path length cuvette.

Resonance Raman spectra were recorded at room temperature and 77 K, the latter with an LN2-flow cryostat (Air Liquide, France). Laser excitations at 476.5, 488.0, 501.7, and 514.5 nm were obtained with an Ar^+^ Sabre laser (coherent). Output laser powers of 10–100 mW were attenuated to <5 mW at the sample. Scattered light was focused into a Jobin-Yvon U1000 double-grating spectrometer (1800 grooves/mm gratings) equipped with a red-sensitive, backilluminated, and LN2-cooled CCD camera. Sample stability and integrity were assessed based on the similarity between the first and last Raman spectra.

## Results

### Absorption of Violaxanthin, Vaucheriaxanthin, and Fucoxanthin

**Figure 2:**
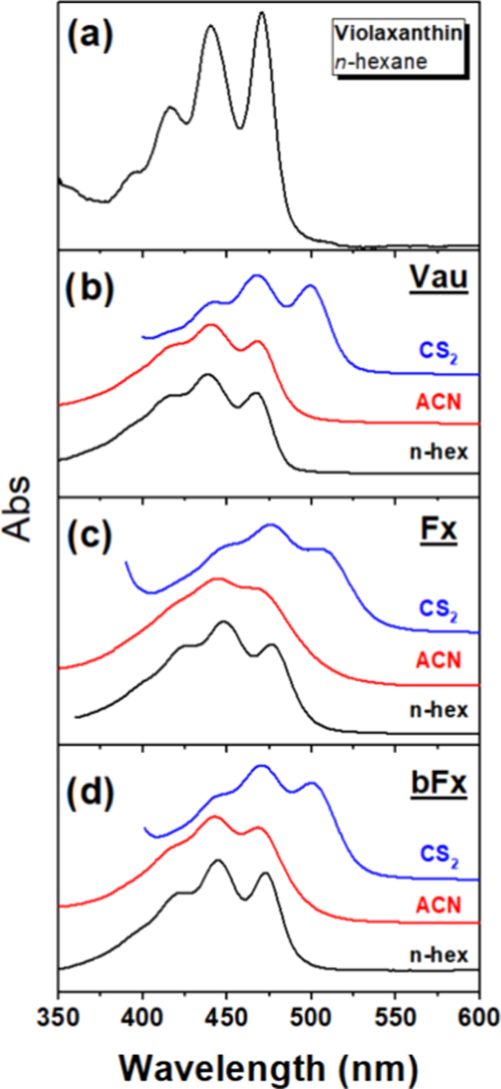
Absorption spectra at room temperature of Vio (a), Vau (b), Fx (c), and bFx (d) in *n*-hexane (black), ACN (red), and CS_2_ (blue). Violaxanthin is chosen for comparison as its chemical structure differs from vaucheriaxanthin only at the level of the allene group.

Figure 2 shows the absorption spectra of Vau, Fx, and bFx at room temperature, in *n*-hexane, acetonitrile (ACN), and carbon disulfide (CS_2_), together with the absorption spectrum of Vio in *n*-hexane for comparison. These absorption spectra are typical for carotenoids, with three clear vibronic peaks referred to as 0–0, 0–1, and 0–2 of the S_0_–S_2_ electronic transition. In *n*-hexane (Table 1), these absorption bands are located at 467.8, 439.0, and 416.0 nm for Vau; 477.0, 448.6, and 423.7 nm for Fx; and 473.2, 444.7, and 420.4 nm for bFx (0–0, 0–1, and 0–2, respectively). The Fx and bFx carotenoids have a broad absorption spectrum in ACN, as already described for carotenoids containing conjugated keto groups.^19,20^

**Table 1:** Values of 0–0 Transition Energy and Resonance Raman Frequencies of the ν_1_ and Most Intense ν_2_ Bands for Vau, Fx, and bFx in Different Solvents^a^

		0–0 (nm)	Ex (nm)	ν_2_ (cm^–1^)	ν_2_ (cm^–1^)	ν_1_ (cm^–1^)	ν_1_ (cm^–1^)
Vau	*n*-hexane	467.8	476.5	1153	1158	1527	1531
	ACN	468.4	476.5	1152	1159	1526	1532
	CS_2_	500.0	501.7	1150	1159	1523	1529
Fx	*n*-hexane	477.3	488.0	1151	1160	1527	1534
	ACN	475.6	514.5	1152	1160	1526	1534
	CS_2_	508.0	488.0	1150	1159	1524	1532
bFx	*n*-hexane	472.9	476.5	1150	1160	1527	1535
	ACN	471.0	476.5	1150	1160	1527	1535
	CS_2_	502.0	501.7	1150	1160	1523	1533

a As these resonance Raman bands contain convolved contributions for all of these carotenoids, this table reports two frequencies for each band, obtained by calculating the second derivative of the spectra.

For comparison, the absorption band corresponds to the 0–0 electronic transition of Vio peaks at 470 nm (21 276 cm^–1^), which is close to the value expected for a 9.0 C=C canonical carotenoid^5^ (e.g., neurosporene in *n*-hexane absorbs at 21–408 cm^–1^, 467 nm). The position of the 0–0 peak of vaucheriaxanthin in *n*-hexane is nearly identical to that of neurosporene (*N* = 9), suggesting that, as expected, the C8′=C7′ bond of the allene group is conjugated with the rest of the chain. Note that the π orbitals of the second allene C=C (between C7′ and C6′) are orthogonal and so do not extend the conjugation further. The 0–0 position for Fx falls in between that of neurosporene and spheroidene (*N* = 10),^5^ closer to spheroidene, even though we would expect *N*_eff_ = 9 in total (eight C=C double bonds, taking C8′=C7′ into account, plus one C=O)—the conjugated carbonyl in Fx appears to extend *N*_eff_ by more than 1. On the other hand, the presence of an unconjugated acyloxy group at the opposite end to this C=O (i.e., in bFx) drags the absorption position of the molecule back toward the blue, midway between neurosporene and spheroidene. It is surprising that this group cancels the additional effect of the conjugated C=O, even though this acyloxy is not part of the conjugated chain.

### Absorption Modeling

**Figure 3:**
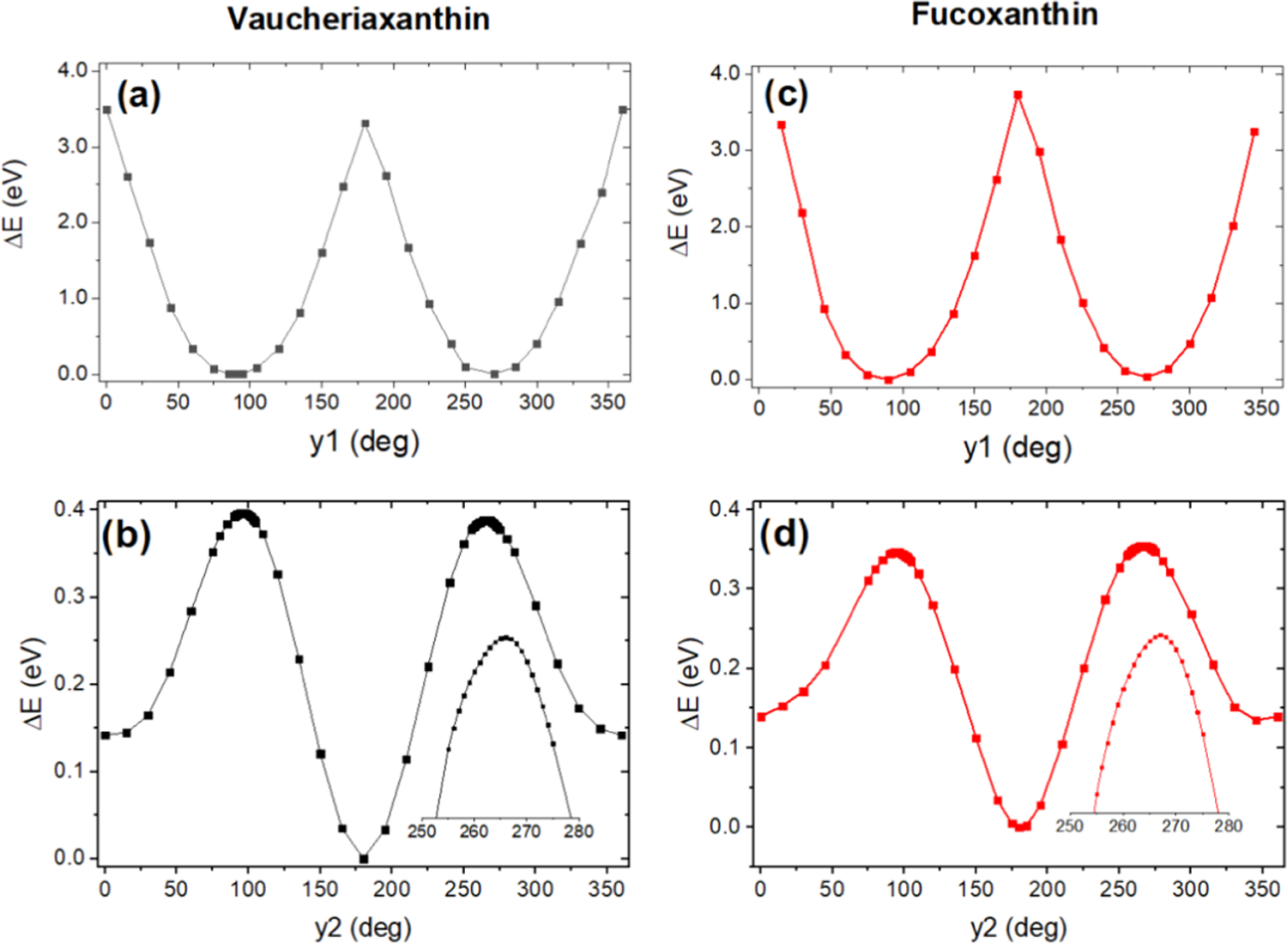
Relative ground-state energies of vaucheriaxanthin (a, b) and fucoxanthin (c, d) according to the end-group and polyene chain position, upon rotation in dihedral angles γ_1_ (a, c) and γ_2_ (b, d) from the minimized starting conformations.

To understand the parameters underlying the absorption of allene carotenoids, we performed extensive quantum chemical calculations on both vaucheriaxanthin and fucoxanthin. Despite their apparent simplicity, the isoprenoid structure of carotenoids renders a full calculation of their electronic structure somewhat complex. However, this has become increasingly possible, thanks to the development of DFT and TD-DFT methods, and their combination with QM/MM calculations or Car–Parrinello molecular dynamics (CPMD).^21−32^ It is of note that, for some carotenoids (e.g., those containing conjugated alkyne groups), the energy barriers to rotation around particular C≡C bonds are low enough so that nonminimum conformations become dynamically populated in solution at room temperature. The empirical properties of alkyne carotenoids cannot be fully modeled without taking into account the dynamic presence of these conformers. Following a similar strategy, we looked for conformers possessing different configurations of the allene group by rotating the two possible dihedral angles, γ_1_ and γ_2_, involving this group. γ_1_ is the dihedral angle corresponding to rotations around the double C7′–C8′ bond (see Figure 1). As this bond is a C=C, the energy associated with this rotation is expected to be high. γ_2_ is the dihedral angle corresponding to rotations around the single C8′–C9′ bond (see Figure 1). These two dihedral angles control the in-plane position of the allene group and more generally of the whole terminal end of these carotenoids. These two rotation angles were used to look for local minima of Vau and Fx. The end-group potential energy surfaces were calculated by finding the global minimum, and the dihedral angles, first γ_1_ followed by γ_2_, were then changed stepwise from 0 to 360°, and the ground-state energy was calculated for each step (Figure 3). In this approach, by generating starting geometries, an energy map is created containing information on the rotation energy barriers, and, at the same time, it may reveal local minima for metastable configurations.^11^ These energy surface studies did not reveal any obvious candidates for such metastable conformations—the optimization *in vacuo* of all generated conformers evolved toward the global minima for angles γ_1_ or γ_2_.

**Figure 4:**
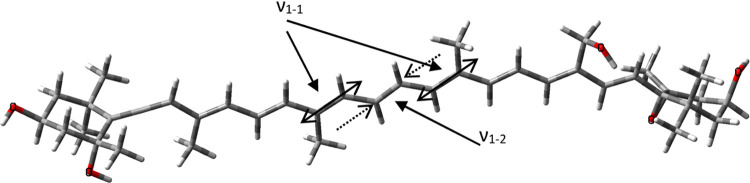
Vaucheriaxanthin structure: polyene chain on the left side is extended by the allene group. Arrows indicate the two major C=C bond stretching vibrations, ν_1–1_ and ν_1–2_.

All*-trans* structures of Vio, Vau, Fx, and bFx were optimized using density functional theory (DFT), and the absorption spectra were recalculated using time-dependent density functional theory (TD-DFT). The starting Vio and Vau structures were taken from PubChem. Two structures were chosen for Fx: an artificially made all-*trans* conformer (all-*trans*-Fx) and the model from PubChem CID 5281239 (Fx_cid_). bFx was constructed artificially from the all-*trans*-Fx optimized structures. The optimization procedure was first performed *in vacuo*, and the structures were then recalculated in *n*-hexane, acetonitrile, and CS_2_ using the polarizable continuum model (PCM) as implemented in Gaussian 16 (Gaussian Inc., Wallingford CT). Calculations using B3LYP/aug-cc-pVDZ and B3LYP/aug-cc-pVTZ showed various convergence problems, while those using cc-pVDZ with B3LYP or CAM-B3LYP were generally successful. This calculation level is quite accurate but necessitates taking into account scaling factors between 0.9 and 0.999 to obtain results comparable with experimental data (the exact scaling factor depending on the precise nature of the carotenoid). For the purposes of this work, the scaling factor was not applied except where a direct comparison was given with the experimental data. The minimized structure of vaucheriaxanthin *in vacuo* is given in Figure 4 as an example.

Table 2 displays the results obtained following four distinct procedures to calculate the first optically allowed excited state of vaucheriaxanthin. In the first procedure, we optimized the Vau structure at the B3LYP/cc-pVDZ level *in vacuo* and subsequently recalculated the excited state using PCM models at the same level. In the second procedure, the B3LYP/cc-pVDZ-level-optimized structure *in vacuo* was again used to recalculate the excited state with PCM models but this time using the CAM-B3LYP/cc-PVDZ level. In the third and fourth procedures, the optimized structure and excited states were performed using CAM-B3LYP/cc-PVDZ and B3LYP/cc-PVDZ, respectively. To fit the experimental data obtained for this carotenoid in *n-*hexane, ACN, and CS_2_, the first procedure requires scaling factors 0.822, 0.826, and 0.856, respectively. The second procedure overestimates the energy values, dragging these scaling factors to 1.046, 1.05, and 1.099. The third procedure requires scaling factors 0.954, 0.958, and 0.998, while procedure 4 requires factors 0.821, 0.825, and 0.856. For all procedures, the scaling factor required is observed to vary by up to 0.05 for different PCM solvents. Scaling factors must thus be applied very carefully when comparing theoretical calculations using PCM with experimental data.

**Table 2:** Experimental Values of 0–0 Transition Energy for Vau and Results with the Four Different Calculation Procedures

		0–0 (nm)	ε	procedure 1 (nm)	procedure 2 (nm)	procedure 3 (nm)	procedure 4 (nm)
Vau	*n*-hexane	467.8	1.8819	569.36	447.15	490.49	569.51
	ACN	468.4	35.688	567.14	446.02	489.08	567.42
	CS_2_	500.0	2.6105	583.91	455.03	500.94	584.11
	vacuum			532.93	463.89	426.79	532.93

Similar difficulty is observed when comparing the calculated values in a given solvent for the different carotenoid molecules. The measured absorption position of Vau, Fx, and bFx in *n*-hexane is 467.8, 477.3, and 472.9 nm, respectively, while the calculated values using procedure 3 as an example are 490.49 nm (Vau), 509.17 nm (Fx), and 484.97 nm (bFx). The scaling factors required to fit the experimental values would thus be 0.954, 0.937, and 0.975. Using the same procedure thus does not guarantee the same scaling factors between experimental and calculated values for different carotenoids, as already shown when using B3LYP/cc-pVDZ.^15^ We thus decided to analyze the results of the modeling of the different carotenoids separately to understand their background properties without directly comparing their experimental values to the calculated ones.

**Figure 5:**
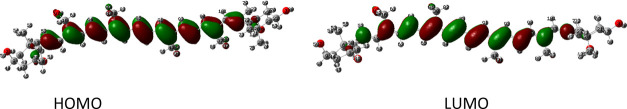
Vaucheriaxanthin HOMO and LUMO orbitals, indicating that the allene group contributes an additional π orbital to the conjugated polyene chain so that *N* = 9.

In the following, we will first consider the computations performed by procedure 4. We first performed control calculations on neurosporene (Neu), which possesses 9 C=C double bonds (*N* = 9) in the polyene chain, and the calculated value for the optical transition is 531.19 nm. If the calculation is performed on spheroidene (N = 10), the optical transition is obtained at 552.57 nm. Violaxanthin (Vio) has different end groups in comparison with Neu, resulting in a calculated value for the optical transition of 534.86 nm. Experimentally, the absorption position of violaxanthin does indeed peak at a somewhat lower energy than neurosporene: 470 nm for Vio vs 466 nm for Neu in *n*-hexane. The calculated optical transition of vaucheriaxanthin peaks at 532.93 nm between the values of Vio and Neu and also in line with the experimental data. These calculations indicate that, as expected, the first C8′=C7′ of the allene group extends the conjugation of Vau to 9 C=C bonds (Figure 5).

For Fx, the calculated value of the optical transition is at 528.40 nm. Similarly, the calculated position of the optical transition for 19′-butanoyloxyfucoxanthin (bFx), which differs from Fx only by the addition of a nonconjugated group, is 525.70 nm. From these calculations, the optical transitions of Fx and bFx should be located almost at the same position as the Vau transition, possibly slightly on the blue side of it. This is not what is observed experimentally, as the position of the optical transition of Fx is close to that of spheroidene and that of bFx halfway between spheroidene and neurosporene. Thus, it appears that the effect of the presence of the electron-rich oxygen atom is only partially accounted for by this level of calculation although it predicts a slight blue shift induced by the presence of the nonconjugated acyloxy group. Procedure 3, which includes long-range corrections, predicts the right position of the Fx absorption on the red side of Vau (509 nm for Fx vs 490 for Vau). However, this same approach yields a too large acyloxy-induced blue shift for bFx, predicting the absorption of the latter carotenoid at 484 nm. Introducing long-range corrections in the calculations thus seem to overestimate the influence of the oxygen atoms present in the carotenoid structure.

Previously, the complex behavior of carbonyl carotenoids such as Fx has been addressed using a hybrid DFT and multireference configuration interaction (MRCI) approach, leading to the conclusion that meta-GGA functionals, particularly, M11L or MN12L, give the best results for DFT modeling of such carotenoids.^33^ The main conclusion was that the C=O moiety reduces the energy of *n*π* transitions such that they become closer to the ππ* transition, which in turn alters the conjugation properties of Fx. Using this procedure, we obtained calculated values of 579.09 nm for Fx and 599.33 nm for Vau when using the M11L functional with the cc-pVDZ basis set, while these values were 552.28 and 561.41 nm, respectively, with the MN12L functional. Thus, in both cases, the energy of the excited states was predicted as Fx < Vau, still the opposite of the empirical relationship. We therefore checked several other functionals and basis set combinations. The PBE1PBE functional predicted 509.48 nm for Fx and 515.22 nm for Vau. The MN12SX functional predicted 521.87 nm for Fx and 528.44 nm for Vau. The M11 functional predicted 579.09 nm for Fx and 587.22 nm for Vau. We also tried to use the B3LYP/cc-pVDZ calculation level for the carotenoid structure and a more accurate basis set for the oxygen (aug-cc-pVDZ), and we still got the prediction that the energy level of the allowed transition should be lower for Fx than for Vau (530.96 nm vs 533.48 nm). On the other hand, a different result is obtained upon perturbation of the conjugated C=O with a hydrogen bond. We optimized a supermolecule containing Fx with one or two water molecules H-bonding its C=O and then calculated the energy of the allowed transition of this supermolecule using the B3LYP/cc-pVDZ calculation level. In the absence of a water molecule, the length of the C=O bond in the *cis* and *trans* position is 1.2237 and 1.2245 Å, and the transition is predicted at 528.40 and 527.95 nm, respectively. Keeping the C=O bond in *trans*, the presence of one water molecule shifted the transition to 535.86 nm and the length of the C=O bond to 1.2314 Å. The presence of two water molecules shifted the transition to 542.68 nm and the C=O distance to 1.2323 Å. With the same calculation level, the Vau transition peaks at 532.93 nm, thus predicting the absorption of Fx to the red of that of Vau, as observed experimentally. As hydrogen bonds result in a stronger conjugation of the C=O group, these calculations suggest that the actual conjugation of this group to the carotenoid C=C chain is underevaluated in these DFT calculations.

### Resonance Raman of Violaxanthin, Vaucheriaxanthin, and Fucoxanthin

To fully understand the effect of the allene groups on the carotenoid properties, it is necessary to characterize their influence on the carotenoid configuration. Resonance Raman spectroscopy, as a vibrational method, yields a detailed picture of this configuration. Carotenoids being among the strongest resonance Raman scatterers among organic molecules, recording their resonance Raman spectra is relatively straightforward. Moreover, as a resonance technique, this approach is highly selective and may help in identifying different configurations present in a given sample.^34,35^ The spectra obtained yield precise information on the effective conjugation length of these molecules through the frequency of the C=C stretching mode (contributing to the spectra as the so-called ν_1_ band in the 1510–1540 cm^–1^ range).^36^ Information on their precise isomerisation state can be drawn from the analysis of their C–C stretching mode contributions (present as a cluster of bands termed ν_2_, 1110–1160 cm^–1^ range).^37−39^

**Figure 6:**
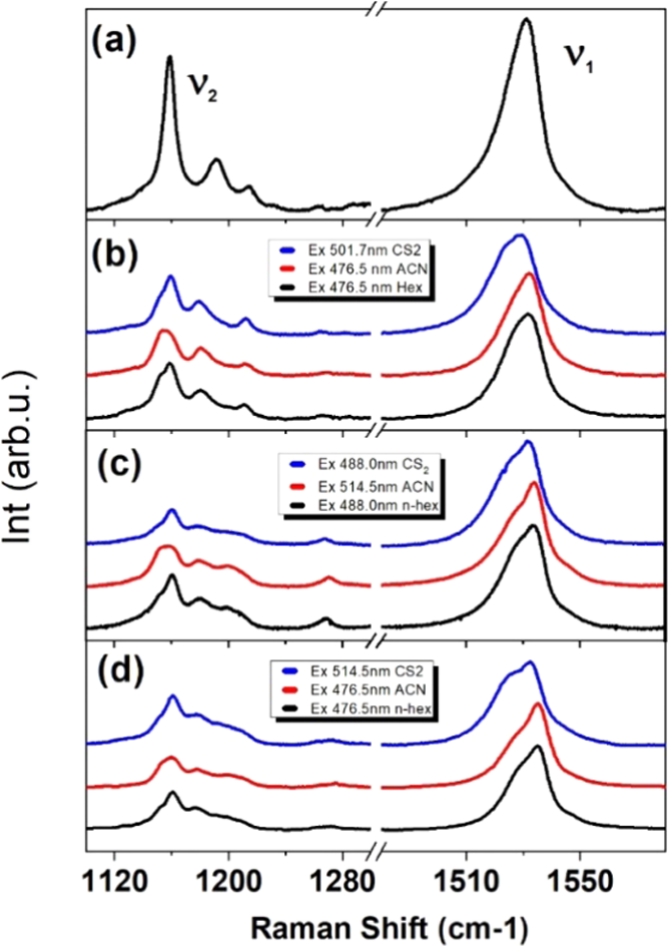
Room temperature resonance Raman spectra of vaucheriaxanthin (b), fucoxanthin (c), and butanoylfucoxanthin (d) in different solvents (CS_2_, ACN, and *n*-hexane), compared to violaxanthin in *n*-hexane (a). The resonance conditions were adapted for each solvent, such that the excitation wavelength corresponded to the peak of the 0–0 sublevel of the S_0_/S_2_ transition.

Figure 6 displays the resonance Raman spectra of Vio, Vau, Fx, and bFx at room temperature. The first striking difference between the spectra of the allene-containing carotenoids and Vio concerns the ratio between the v_1_ and v_2_ bands. In the Vio spectrum, this ratio is quite typical for carotenoids excited in resonance conditions with the 0–0 sublevel of the absorption transition. For allene carotenoids, this ratio is about twice that of Vio (i.e., their v_1_ is twice the size, relative to v_2_). Changes in this ratio may be induced by differences in resonance conditions but that cannot be the case here—all excitations were chosen to be on top of the 0–0 sublevel of the S_0_/S_2_ transition. Indeed, this abnormal ratio has already been observed for Vau when comparing its 77 K resonance Raman spectra with those of diadinoxanthin and heteroxanthin (Figure S1).^40^

In Raman spectra of Vio in *n*-hexane, the ν_2_ region displays a single main peak at 1158 cm^–1^, together with two satellite bands at 1192 and 1215 cm^–1^. This region constitutes a fingerprint for the carotenoid isomerization state (cis/trans),^37−39^ and such a profile unambiguously indicates that violaxanthin is in the all-*trans* configuration. For Vau, Fx, and bFx, the main vibrational mode in this region appears as a doublet. Such a spectral signature has never been reported for any other carotenoid molecule, and so, its origin is difficult to predict. The satellite bands are at similar frequencies for Vau, Fx, and bFx at 1180 and 1211 cm^–1^. Again, the presence of only three bands in this region rules out the possibility of *cis* isomers in the samples. Finally, for violaxanthin, the ν_1_ band, arising from C=C stretching modes, contributes as a single component at 1531.0 cm^–1^ (width 14 cm^–1^, FWHM). It is, however, slightly asymmetric, accompanied by a low-intensity component on the higher frequency side. This mode appears as a doublet for Vau, Fx, and bFx in all solvents used and is 20% broader than observed for Vio. In n-hexane, this double band is centered at 1532.9, 1534.7, and 1535.7 cm^–1^ for Vau, Fx, and bFx, respectively. Decomposing this band into two components introduces some uncertainty about their position (up to 2 cm^–1^, depending on the method used). The frequencies reported in Table 1 have all been determined using the same method, based on the second derivative of the spectra.

**Figure 7:**
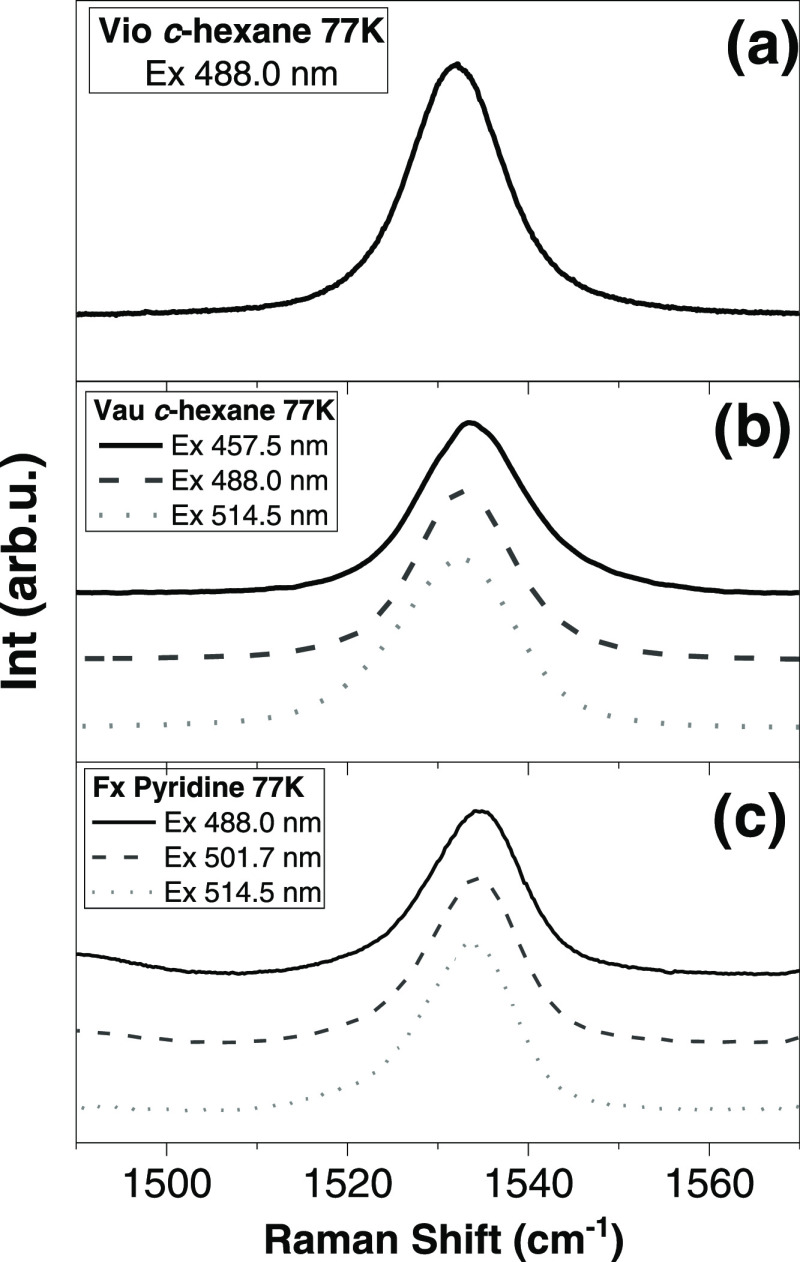
77 K resonance Raman spectra in the ν_1_ region of violaxanthin in *n*-hexane excited at 488.0 nm (a), vaucheriaxanthin in *n*-hexane excited at 457.5, 488.0, and 514.5 nm (b), and fucoxanthin in pyridine excited at 488.0, 501.7, and 514.5 nm (c).

Doublet components in the ν_1_ or ν_2_ bands may be indicative of the presence of a mixture of conformers in the samples, as observed for alkyne carotenoids.^11^ The selectivity of the resonance Raman technique means that such conformers can be distinguished by playing with the energy of the excitation laser beam, as they should exhibit small differences in their absorption properties. This selectivity increases at low temperatures due to the narrowing of the absorption bands used to induce the resonance effect. Figure 7 shows the 77 K resonance Raman spectra of Vio in *n*-hexane excited at 488.0 nm, Vau in *n*-hexane excited at 457.5, 488.0, and 514.5 nm, and Fx in pyridine excited at 488.0, 501.7, and 514.5 nm. At room temperature, the 0–0 electronic transition of Vau in *n*-hexane and Fx in pyridine is at 467 and 496 nm, respectively. We used three different excitation wavelengths to induce resonance Raman spectra, straddling both sides of the 0–0 transition for each allene carotenoid. In these conditions, the presence of different conformers with slightly different absorption properties would result in excitation-dependent changes in the frequency and/or shape of the ν_1_ band. The contribution of red-absorbing conformers (with lower v_1_ frequency) should be favored when the excitation is on the red side of the transition and disfavored when excitation is to the blue of this transition. The ν_1_ mode for Vio appears as a single band peaking at 1528.8 cm^–1^ (FWHM 12 cm^–1^). This band for Vau in *n*-hexane peaks at 1533 ± 0.5 cm^–1^ (FWHM 18 cm^–1^) for excitations at 457.5, 488.0, and 514.5 nm, while Fx in pyridine obtained with 488.0, 501.7, and 514.5 nm excitation exhibits a single band peaking at 1534 ± 0.5 cm^–1^ (FWHM 12 cm^–1^). The constant value for the ν_1_ frequency at different excitation wavelengths suggests that Vau and Fx samples do not contain conformers with changes in their 0–0 transition. It is of note that the ν_1_ doublet disappears at low temperatures, but the width of the ν_1_ band remains a bit larger for the allene carotenoids compared to Vio. The same phenomenon was also observed for the main ν_2_ band (data not shown).

### Modeling the Resonance Raman of Allene-Containing Carotenoids

We performed extensive simulations of Vau and Fx to determine the physical origin of the ν_1_ and ν_2_ doublets in their resonance Raman spectra. Following the conclusions of resonance Raman experiments, only conformers possessing their carbon–carbon backbone in *all-trans* configuration were modeled.

We explored the vibrational properties of different stable conformers in solvents using three different approaches (i) *in vacuo*, (ii) *in solvent*, using a polarizable continuum model (PCM) approach, and (iii) using a supermolecular approach, where solvent molecules are explicitly present around the carotenoid molecule to “lock” it in a given configuration.^11^ We compared the total energies in vacuum of 4 different types of Vau conformer, corresponding to the different minima of the γ_1_ and γ_2_ dihedral angles (Figure 1). All-*trans*-Vau (γ_2_ ≈ 180°) had the same total energy whether the γ_1_ dihedral angle was 269° (end pos2) or 90° (end pos1). For s-cis-8′-Vau (γ_2_ ≈ 0°), the energy was 0.05 eV larger for γ_1_ = 269° (end pos2) and even larger for γ_1_ = 90° (0.11 eV; end pos1). Similar numbers were calculated when the modeling was performed using PCM with *n*-hexane. Table 3 displays the full set of results for these Vau conformers.

**Table 3:** Calculated Resonance Raman Intensities of C=C and C–C Symmetric and Antisymmetric Vibrations for the Different Vaucheriaxanthin Conformers under Various Conditions

vaucheriaxanthin conformer										
γ_1_, deg	γ_2_, deg	ν_1–1_, cm^–1^	Int_1–1_, %	ν_1–2_, cm^–1^	Int_1–2_, %	ν_2–1_, cm^–1^	Int_1–1_, %	ν_2–2_, cm^–1^	Int_2–2_, %	conditions
Calculations for the Different Conformers Using When γ_2_ ≈ 0° as a Starting Point										
89	0	1570	100	1582	23	1200	16	1195	18	vacuum
90	0	1569	100	1581	29	1199	21	1195	17	PCM/*n*-hexane
85	331	1572	100	1581	30	1199	33	1193	1	vacuum
85	333	1571	100	1581	42	1200	31	1194	3	PCM/*n*-hexane
87	337	1567	100	1577	50	1197	31	1186	4	PCM/water
89	336	1571	100	1581	98	1200	30	1191	11	explicit solvent: eight water molecules around allene group (Figure S2)
269	2	1570	100	1581	26	1201	21	1196	8	vacuum
270	0	1569	100	1580	31	1201	27	1191	11	PCM/*n*-hexane
Calculations for the Different Conformers Using When γ_2_ ≈ 180° as a Starting Point										
269	179	1571	100	1583	26	1201	21	1194	14	vacuum
269	179	1569	100	1582	36	1201	24	1192	12	PCM *n*-hexane
90	181	1571	100	1582	23	1201	23	1195	12	vacuum
90	180	1570	100	1582	40	1202	32	1194	13	PCM in *n*-hexane
83	197	1570	100	1582	25	1200	31	1198	23	explicit solvent

**Figure 8:**
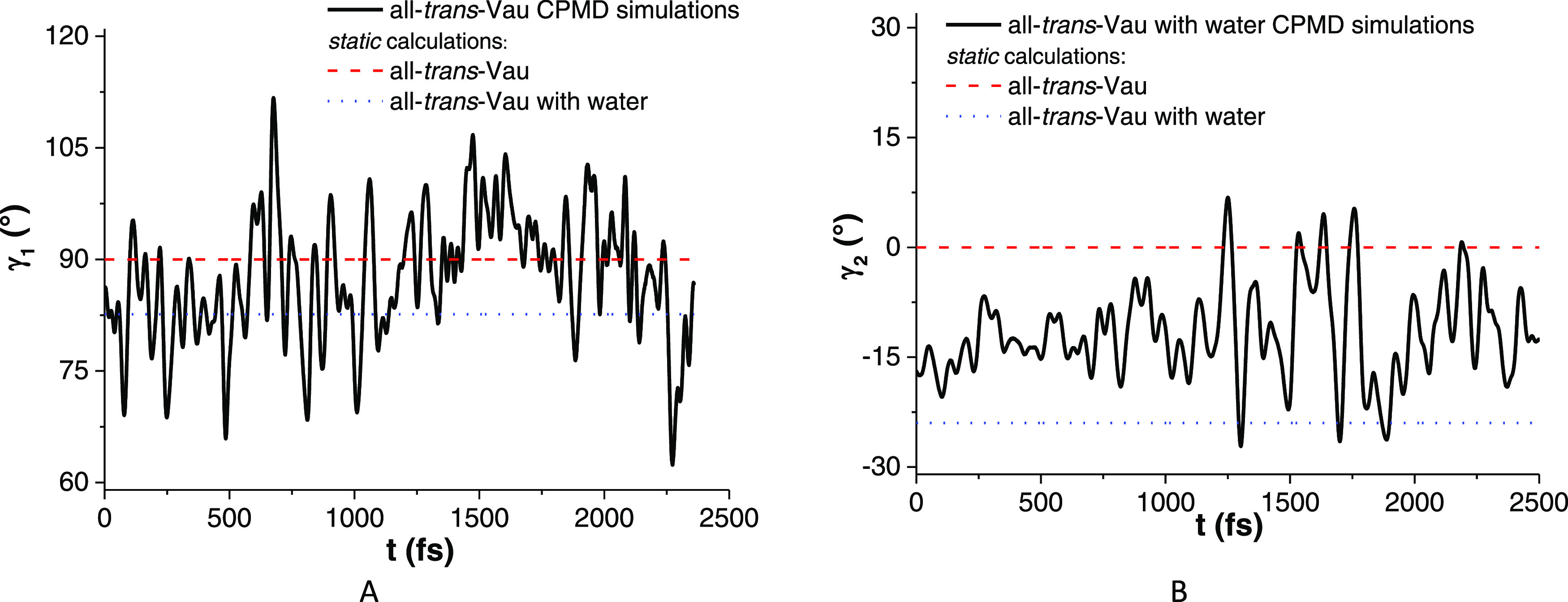
2.5 ps Car–Parrinello molecular dynamics (CPMD) simulations at 300 K: evolution of γ_1_ (left) and γ_2_ (right angles). Dash and dot lines represent the optimized minima obtained from static calculations (see text).

In calculations performed in the simplest conditions (*in vacuo*), we observe that the intensity of the C=C antisymmetric stretching mode is about 25% that of the equivalent symmetric stretch, while the frequency of this antisymmetric C=C is about 10 cm^–1^ higher than the symmetric one. This is in marked contrast to the calculated modes for simpler carotenoids like lutein, neurosporene, and spheroidene. For these nonallene carotenoids, we calculated the position of the symmetric and antisymmetric C=C stretching modes and found that they are also about 10 cm^–1^ apart, but their intensity ratio between them is close to 10, under all conditions calculated (vacuum, various PCM). When the calculation is performed on Vau using the PCM approach, the intensity of the band arising from antisymmetric C=C stretching modes increases, and the ratio between the antisymmetric/symmetric C=C stretching mode reaches 30% in *n*-hexane and 50% in water (Table 3 and Figure S2). When a supermolecular approach is used to explore the effect of nonminimum configurations around the allene group, the intensity of the antisymmetric C=C mode may become even higher, reaching that of the symmetric C=C mode for some conformers. In the ν_2_ region, the same phenomenon occurs, with the antisymmetric C–C stretching modes gaining intensity (although to a lesser extent; Table 3). It is worth noting that while the antisymmetric C=C stretching mode possesses a frequency higher than the symmetric one, the antisymmetric C–C stretching mode is located at lower frequencies than its symmetric counterpart. Analysis of the various Vau configurations shows that, although the antisymmetric C=C stretching mode is already active in the fully relaxed state of this molecule, its intensity is somewhat dependent on deviations of the γ_2_ angle, which bring the whole allene group slightly out of the plane of the C=C conjugated chain. Indeed, a 30° out-of-plane deviation of this group results in a similar contribution to the Raman spectra of both the symmetric and antisymmetric stretching modes. While the lowest energy configuration of Vau was calculated as *all-trans* in all conditions, these distorted configurations were only up to 0.13 eV higher, and most of the time much less. It is thus conceivable that some of these calculated structures, close to the all-*trans* or s-*cis*-8′ configuration, may exist at room temperature and therefore contribute to the measured spectra in these conditions. We looked for these conformers using procedure 4 calculations in the presence of water molecules using PACKMOL software^41^ for optimizing the initial configurations for molecular dynamics simulations and removing stepwise the water molecule during the trajectory. We run several Car–Parinello molecular dynamics (CPMD) simulations to additionally check the possibility of the presence of such structures at room temperature, using the NwChem program (version 7.0.2).^42^ These calculations were performed at 300 K, with a time step of 3.0 au (0.07257 fs), coupled to a Nosé–Hoover chain thermostat^21^ at a frequency of 1200 cm^–1^, and an electronic mass parameter was 450 au. Electronic exchange and correlation were modeled using the gradient-corrected functional of Perdew, Burke, and Ernzerhof (PBE).^22^ Core electrons were treated using the norm-conserving atomic pseudopotentials (PP) of Troullier and Martins^23^ while valence electrons were represented in a plane-wave basis set truncated at an extended energy cutoff of 20 Ry. Following the initial equilibration period, the γ_1_ and γ_2_ trajectories suggest that the distorted configurations may appear involving both the dihedral angles γ_1_ and γ_2_ and can reach those predicted by the static calculations (Figure 8).

**Figure 9:**
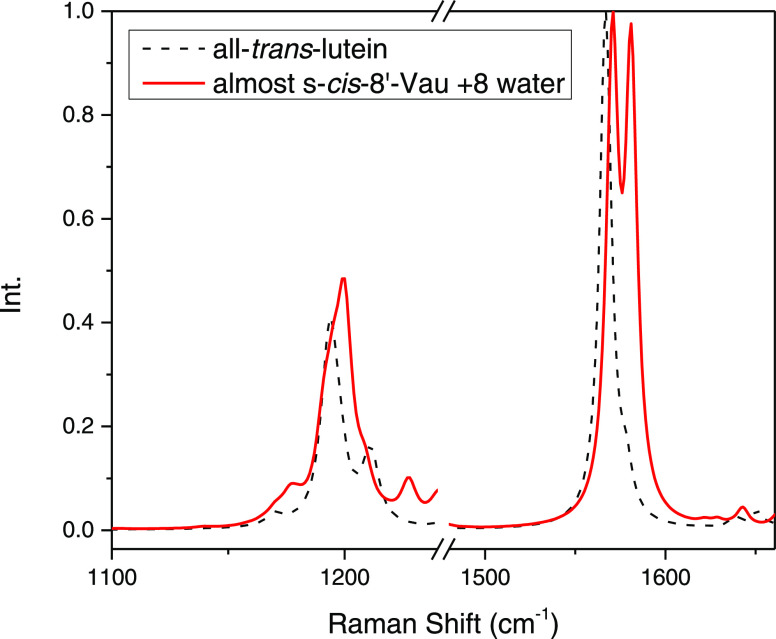
Comparison of calculated resonance Raman spectra of vaucheriaxanthin and lutein, showing spectroscopic consequences of the gain in the intensity of the C=C and C–C antisymmetric stretching modes.

As an illustration of the consequences of the intensity gain of the C=C asymmetric stretching mode on the resonance Raman spectra of Vau, Figure 9 shows the comparison of the calculated resonance Raman spectra of vaucheriaxanthin and lutein. The gain in the intensity of the antisymmetric stretching modes in the case of Vau induces the presence of two convolved components in the main band of the ν_2_ region and two clear components in the ν_1_ region. It is of note that, due to broadening, both the components in the ν_1_ region will merge, resulting in the presence of a convolved band of almost double intensity in this region, exactly as observed experimentally (see Figure 6). It is worth noting that, as the gain in the intensity of the antisymmetric stretching modes is enhanced by the presence of conformers with higher ground-state energies, our calculations predict that the intensity of the band arising from these modes should decrease when lowering the temperature, in line with the experimental results.

**Table 4:** Calculated Resonance Raman Intensities of C=C and C–C Symmetric and Antisymmetric Vibrations for the Different Fx Conformers under Various Conditions

Fx structure											
γ_1_, deg	γ_2_, deg	γ_3_, deg	ν_1–1_, cm^–1^	Int_1–1_, %	ν_1–2_, cm^–1^	Int_1–2_, %	ν_2–1_, cm^–1^	Int_1–1_, %	ν_2–2_, cm^–1^	Int_2–2_, %	PCM solvent*
272	359	16	1568	100	1582	27	1211	4	1195	26	vacuum
272	359	12	1563	100	1582	29	1211	8	1195	16	PCM/*n*-hexane
87	349	15	1567	100	1582	26	1207	3	1195	22	vacuum
90	355	16	1564	100	1582	27	1207	5	1195	21	PCM/*n*-hexane
270	180	16	1569	100	1584	28	1205	7	1198	20	vacuum
271	180	15	1566	100	1583	35	1207	11	1199	20	PCM/*n*-hexane
88	181	12	1568	100	1583	27	1206	8	1198	16	vacuum
90	180	18	1565	100	1582	30	1206	6	1198	24	PCM/*n*-hexane
88	183	179	1571	100	1583	26	1202	17	1199	16	vacuum
91	181	180	1569	100	1582	26	1204	17	1199	15	PCM/*n*-hexane
89	181	178	1573	100	1584	31	1205	13	1198	8	vacuum
90	181	178	1570	100	1582	32	1204	22	1199	10	PCM/*n*-hexane
270	180	178	1573	100	1584	31	1203	19	1198	9	vacuum
270	180	179	1571	100	1583	33	1204	21	1199	7	PCM/*n*-hexane
271	359	178	1572	100	1583	28	1205	15	1194	20	vacuum
88	351	177	1572	100	1583	29	1205	17	1194	19	vacuum

Calculations performed on fucoxanthin in the same conditions lead to similar results (Table 4). It is worth mentioning that Fx may exhibit additional specific conformers corresponding to different values of the γ_3_ dihedral angle, around the C8–C9 bond, on the opposite end of the molecule with respect to the allene group. Such conformers could, in turn, influence the vibrations of the polyene chain. However, the detailed analysis did not reveal any significant dependence of the symmetric and antisymmetric stretching intensities on this dihedral angle.

## Discussion

In this work, we have attempted to model the electronic and vibrational properties of different allene carotenoid molecules, one containing only an allene group (Vau) and two containing a conjugated carbonyl group in addition (Fx and bFx). Modeling can satisfactorily explain the electronic properties of vaucheriaxanthin, where the allene group introduces the equivalent of one C=C double bond into the conjugated C=C chain. It is worth noting that the π-orbitals of the two C=C bonds of the allene group are necessarily orthogonal to each other so that only one can be involved in the conjugated chain. Thus, the allene group *per se* is not fully conjugated, as concluded earlier, from the absence of typical allene frequencies in the resonance Raman spectra of fucoxanthin.^18^ We needed to perform careful analysis separately for the absorption peak positions and Raman assignments of the selected carotenoids (Cars), which required different DFT methodologies as well.

It is worth noting that attempts to model carotenoids containing a conjugated carbonyl group have failed in the past. For instance, the absence of clear 0–0, 0–1, and 0–2 sublevels in the absorption spectra of spheroidenone (which is a molecule equivalent to spheroidene with an additional conjugated C=O at the end of the C=C chain) could not be predicted by calculations.^42^ Thus, in the present state of DFT calculations, the presence of a conjugated C=O bond in the carotenoid structure remains a challenge to modelers. The position of the electronic absorption of Fx and bFx is thus expected to be complex to predict properly. In most of the calculation levels, we tried to predict this absorption to lie on the blue side of that of vaucheriaxanthin (rather than to the red, as observed experimentally). However, correct fucoxanthin absorption position (lying on the red side of that of vaucheriaxanthin) can be obtained either by adding H bonds to its conjugated C=O, or taking into account, long-range corrections (as in procedure 3). The latter approach sounds more satisfying, however, it tends to overestimate the role of the nonconjugated acyloxy group in butanoyloxyfucoxanthin. It correctly predicts, in line with the experimental results, that the absorption position of this molecule should be blue-shifted, but the extent of the predicted acyloxy-induced blue shift is 4 times larger than experimentally observed.

A striking property of the allene-containing carotenoids is that they display a resonance Raman spectrum, where the main band arising from both the C=C and the C–C stretching modes is split into two components. A similar effect was reported for carotenoids containing a conjugated alkyne group, but in this latter case, only the band arising from the C=C stretching mode exhibited two components. We showed that this splitting was probably due to the presence of several conformers in equilibrium at room temperature. In the present case, the presence of such conformers in allene-containing carotenoids can be ruled out by analysis of low-temperature resonance Raman spectra, where the frequency of the ν_1_ mode remains constant at all excitation energies. Calculations performed in all conditions, *in vacuo*, by PCM, or using a supermolecular approach, suggest that the resonance Raman of these carotenoids should contain higher contributions from the antisymmetric modes in the ν_1_ and ν_2_ regions than usually observed for simple carotenoids. In carotenoids like lutein, for instance, the antisymmetric stretching mode intensity is low enough so that the corresponding band is barely observed, resulting in only a slight asymmetry of the band arising from the symmetric C=C stretching mode. By contrast, calculations for allene carotenoids suggest that the intensity of the C=C antisymmetric stretching mode is at least 30% that of the symmetric stretching mode, and it may, in some cases, reach a similar intensity. As the corresponding increase of the C–C antisymmetric stretching mode is less pronounced, this results in a global increase of the Raman contributions in the ν_1_ region, which is actually observed experimentally. It is worth noting that, as predicted by the calculations, the low-intensity antisymmetric C–C stretching mode appears in the experimental spectra on the low-frequency side of the main ν_2_. Hence, we conclude that the doublets observed in the ν_1_ and ν_2_ regions arise from an increase in the intensity of the antisymmetric mode contributions in these regions due to the presence of the conjugated allene group.

Given that the antisymmetric C=C stretching mode is the highest frequency component of the v_1_ band, the symmetric C=C stretching mode for the three molecules studied here must be located at around 1527 cm^–1^ for the carotenoid in *n*-hexane. Such a frequency is close to that observed for lutein, which has an effective conjugation length of 9.3. However, the 0–0 absorption transition of lutein in the same solvent is located at 472 nm, while it is at 467, 477, and 472 nm for Vau, Fx, and bFx, respectively. This could suggest that these three molecules deviate from the linear relationship between absorption position and ν_1_ frequency, usually observed for simpler carotenoid molecules. It should be emphasized, however, that these differences are at the limit of the errors observed when comparing the experimental data with the theoretical curve (see Figure S3). The presence of the same frequency for three differently absorbing carotenoids does nevertheless suggest that the additional conjugated groups induce specific perturbations to the structure of the carotenoid ground or excited states, or both, such that they deviate slightly from the canonical relationship.

## Conclusions

In this work, we report a detailed experimental analysis of the absorption and resonance Raman properties of carotenoid molecules, all containing a conjugated allene group. We show that the presence of this group increases the length of the conjugated chain of these carotenoids by the equivalent of one C=C. Using density functional theory (DFT) modeling, we could satisfactorily reproduce these results. Among the studied carotenoids, fucoxanthin and butanoyloxyfucoxanthin contain a conjugated C=O group, and butanoyloxyfucoxanthin binding in addition a nonconjugated acyloxy group. Both these groups have been reported to dramatically influence the excited state structure of these molecules.^12^ They affect the absorption properties of these carotenoids, the conjugated C=O shifting their absorption to the red, while the nonconjugated acyloxy group partly counterbalancing that effect. We show that taking into account long-range corrections in the calculations allows a qualitative modeling of these effects, although predicting it larger than experimentally observed.

The presence of a conjugated allene group additionally induces a splitting of the two most intense resonance Raman bands of these carotenoids at room temperature. Modeling these vibrational properties using DFT approaches in vacuum and PCM models led to different predictions than using supermolecular models including hydrogen bonding. We report a computational procedure, which allows additional conformers to be included in the modeling of the vibrational properties: we analyzed trajectories of carotenoids obtained using Car–Parrinello molecular dynamics calculations and extracted specific hydrogen bond networks, which could be included in supermolecular computations. This way, we circumvent the weakness of DFT calculations performed in vacuum, and we can include, at least for a part, the effect of intermolecular interactions. Such an approach predicts additional local minima for allene carotenoids, which result in an increase of the resonance Raman activity of the bands arising from the antisymmetric C=C and C–C stretching modes. We show that the experimental splitting of the Raman bands is satisfactorily modeled by such effect. As the content and conformation of carotenoids in proteins are often deduced from the structure of the band arising from these stretching modes, our data may thus prevent misled conclusions to be drawn from such studies, when performed on allene carotenoids.

## Supporting Information Available

Figures of the additional molecular structures of carotenoids mentioned in the main text; figure for the vaucheriaxanthin structure obtained by modeling (procedure 4) in the presence of explicit solvent (eight water molecules); absorption plotted according to Raman ν_1_ frequency for allene carotenoids and compared to carotenoids with simpler chemical structures (PDF)
