# Supplementary material for: Electronic and Vibrational Properties of Allene Carotenoids
Source: J Phys Chem A. 2022 Feb 3;126(6):813–24. doi: 10.1021/acs.jpca.1c09393 (PMC8859822; doi:10.1021/acs.jpca.1c09393)
Supplement: Supplementary file 1 — jp1c09393_si_001.pdf [file jp1c09393_si_001.pdf]

Electronic and Vibrational Properties of Allene Carotenoids

Supplementary material

Mindaugas Macernis[a+], Simona Streckaite[b+], Radek Litvin[c,d], Andrew A. Pascal[b], Manuel J. Llansola-Portoles[b], Bruno Robert[b*], Leonas Valkunas[a,e*],

a) Institute of Chemical Physics, Faculty of Physics, Vilnius University, Saulėtekio Ave. 3, LT-10222, Vilnius, Lithuania
b) Université Paris-Saclay, CEA, CNRS, Institute for Integrative Biology of the Cell (I2BC), 91198, Gif-sur-Yvette, France
c) Biology Centre, Czech Academy of Sciences, Branisovska 31, 370 05 Ceske Budejovice, Czech Republic
d) Faculty of Science, University of South Bohemia, Branisovska 1760, 370 05 Ceske Budejovice, Czech Republic
e) Molecular Compounds Physics Department, Center for Physical Sciences and Technology, Sauletekio Ave. 3, LT-10257, Vilnius, Lithuania

[+]: equivalent authors

[*]: corresponding authors

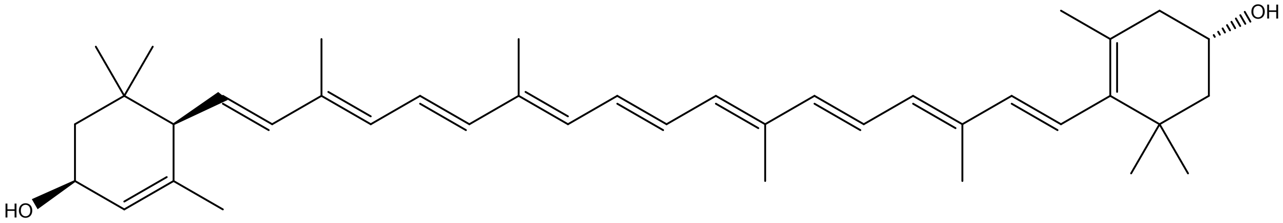

Lutein (N=9+)

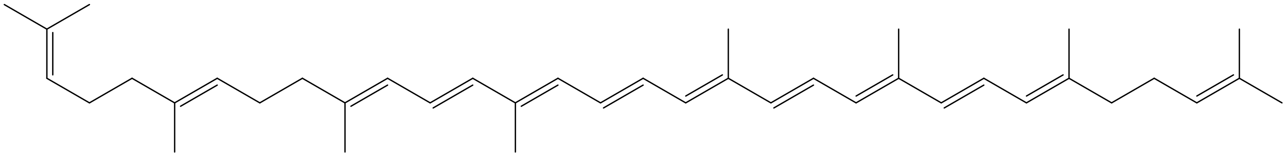

Neurosporene (N=9)

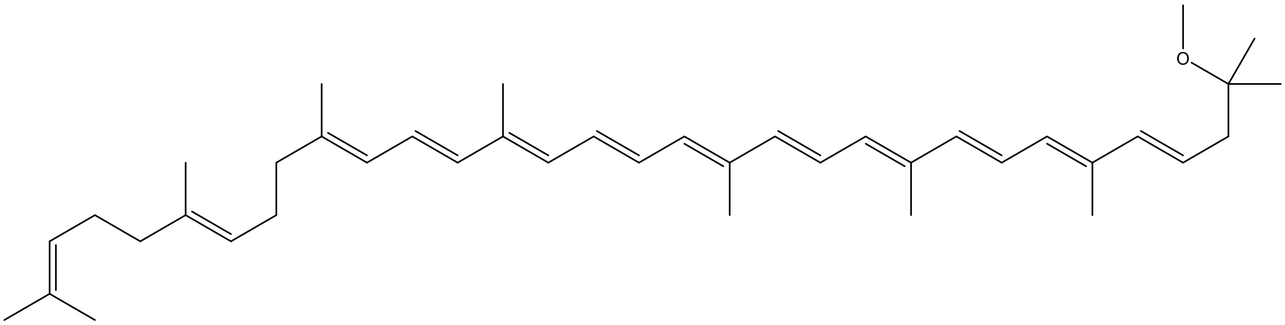

Spheroidene (N=10)

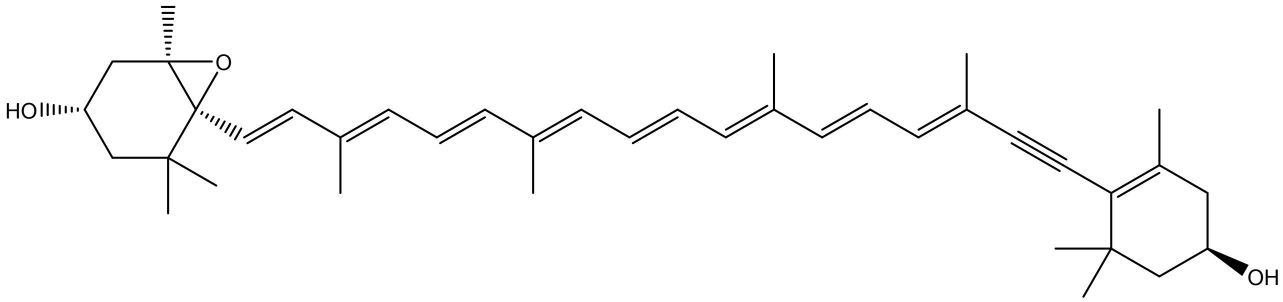

Diadinoxanthin (N=8+)

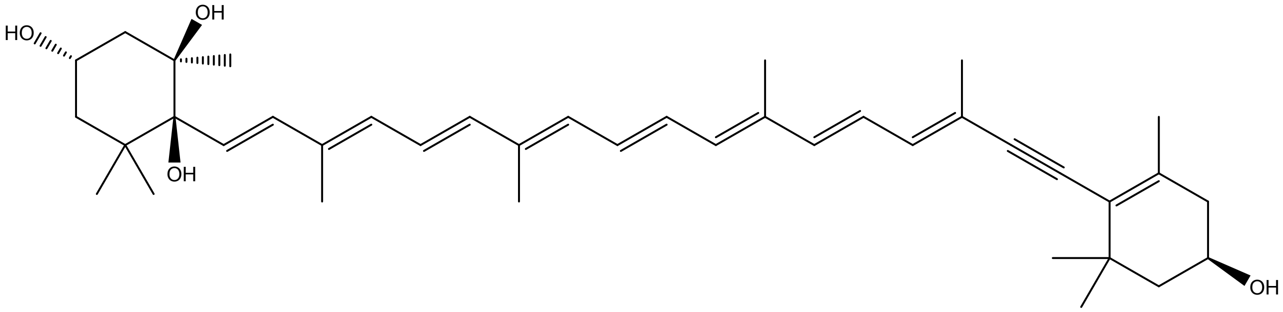

Heteroxanthin (N=8+)

Figure S1/*Additional molecular structures of carotenoids mentioned in the main text.*

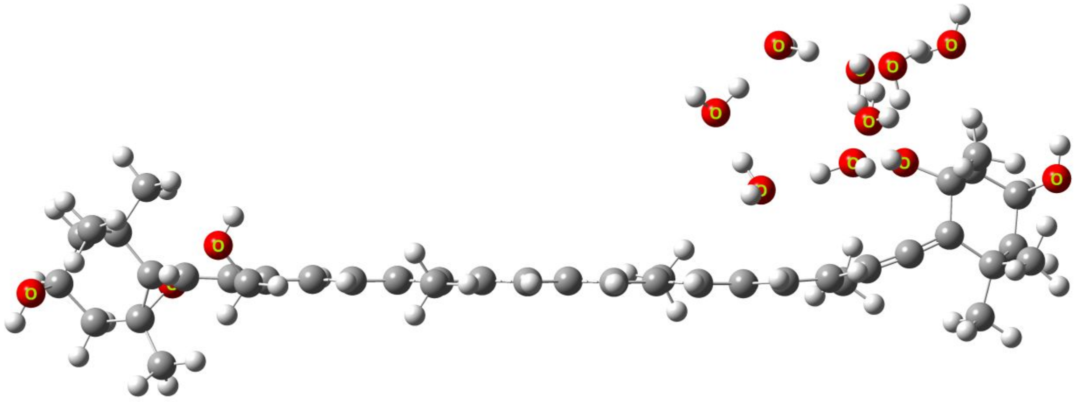

Figure S2/ *Vaucheriaxanthin (all-trans-Vau) structure obtained by modelling (procedure 4) in the presence of explicit solvent (eight water molecules) procedure 4: water molecules were removed one by one till the final complex still reached a non-planar local minima (γ2≠360)*

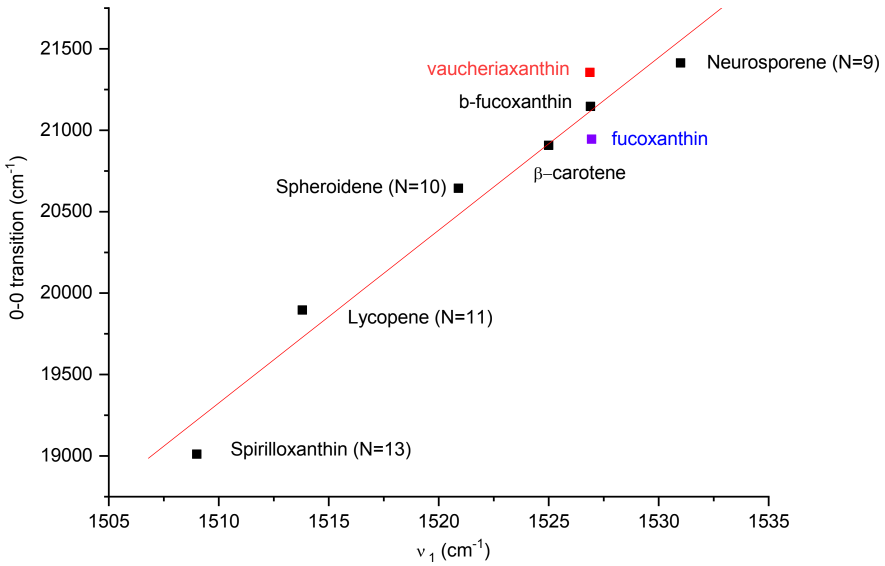

Figure S3/*Absorption (in* cm^{-1}*) plotted according to* v_1 *frequency for allene carotenoids, and compared to carotenoids with simpler chemical structures (replotted from reference 5)*
